# Leg surface electromyography patterns in children with neuro-orthopedic disorders walking on a treadmill unassisted and assisted by a robot with and without encouragement

**DOI:** 10.1186/1743-0003-10-78

**Published:** 2013-07-18

**Authors:** Tabea Aurich Schuler, Roland Müller, Hubertus JA van Hedel

**Affiliations:** 1 Rehabilitation Center Affoltern am Albis, Children’s University Hospital Zurich, Muehlebergstrasse 104, CH-8910, Affoltern am Albis, Switzerland; 2 Children’s Research Center, Children’s University Hospital Zurich, Steinwiesenstrasse 75, CH-8032, Zurich, Switzerland; 3 Institute of Human Movement Sciences and Sport, ETH Zurich, HIT J 32.3, Wolfgang-Pauli-Strasse 27, CH-8093, Zurich, Switzerland

**Keywords:** Surface electromyography, Children, Treadmill, Robotic-assisted gait training, Lokomat®

## Abstract

**Background:**

Robot-assisted gait training and treadmill training can complement conventional physical therapy in children with neuro-orthopedic movement disorders. The aim of this study was to investigate surface electromyography (sEMG) activity patterns during robot-assisted gait training (with and without motivating instructions from a therapist) and unassisted treadmill walking and to compare these with physiological sEMG patterns.

**Methods:**

Nine children with motor impairments and eight healthy children walked in various conditions: (a) on a treadmill in the driven gait orthosis Lokomat®, (b) same condition, with additional motivational instructions from a therapist, and (c) on the treadmill without assistance. sEMG recordings were made of the tibialis anterior, gastrocnemius lateralis, vastus medialis, and biceps femoris muscles. Differences in sEMG amplitudes between the three conditions were analyzed for the duration of stance and swing phase (for each group and muscle separately) using non-parametric tests. Spearman’s correlation coefficients illustrated similarity of muscle activation patterns between conditions, between groups, and with published reference trajectories.

**Results:**

The relative duration of stance and swing phase differed between patients and controls, and between driven gait orthosis conditions and treadmill walking. While sEMG amplitudes were higher when being encouraged by a therapist compared to robot-assisted gait training without instructions (0.008 ≤ p-value ≤ 0.015), muscle activation patterns were highly comparable (0.648 ≤ Spearman correlation coefficients ≤ 0.969). In general, comparisons of the sEMG patterns with published reference data of over-ground walking revealed that walking in the driven gait orthosis could induce more physiological muscle activation patterns compared to unsupported treadmill walking.

**Conclusions:**

Our results suggest that robotic-assisted gait training with therapeutic encouragement could appropriately increase muscle activity. Robotic-assisted gait training in general could induce physiological muscle activation patterns, which might indicate that this training exploits restorative rather than compensatory mechanisms.

## Background

Independent walking is not only a hallmark of motor development [1], it is also one major goal in the rehabilitation of patients with neurological or orthopedic problems to enable independence in functional mobility and daily life activities as well as participation in the society [2,3]. Therefore, huge effort is made to regain independent walking or at least to improve existing but limited walking capabilities. Nowadays, walking over-ground with walking aids or assistance during conventional physiotherapy is being complemented with bodyweight supported treadmill training (BWSTT) with or without therapeutic assistance or robotic-assisted gait training (RAGT) [1,4,5].

There is still little clinical evidence for the efficacy of BWSTT or RAGT in children with neuro-orthopedic gait impairments [6-8].

Nevertheless, RAGT may strengthen neural pathways and enable the nervous system to explore movement variability associated with the production of coordinated locomotion [9]. As practicing over-ground walking is often not possible in children with severe motor impairments, such patients require a simplified and safe therapy environment, such as provided by RAGT, which in addition can provide prolonged training duration with many repetitions of steps, while inducing a reproducible, kinematically consistent, symmetrical gait pattern [10,11].

To investigate whether training modalities such as BWSTT or RAGT might provide the requirements for re-learning to walk in children with neuro-orthopedic problems, it would be of interest to examine whether these modalities could induce appropriate kinematics and/or muscle activation patterns. First, active participation of the child is required to improve walking capability and regain motor function [12,13]. Therefore, an increase in appropriately timed muscle activation would be desired. Second, rehabilitation interventions could be applied that exploit compensatory or restorative mechanisms. Currently, the latter is favored and therefore it would also be of interest to investigate whether RAGT or treadmill walking could induce appropriate muscle activation in children with neuro-orthopedic gait disorders and therefore might exploit more restorative rather than compensatory mechanisms. This issue could also be relevant for engineers to improve robotic rehabilitation technologies.

In a previous study, adult patients with severe hemiparesis after stroke could increase their shortened single support time after 20 session of RAGT to almost normal values, whereas the control group (undergoing 20 sessions of conventional physiotherapy) did not show relevant changes [14]. These results might indicate that RAGT was significantly more effective in recovering the gait pattern than conventional physiotherapy and that improvements in gait symmetry can be transferred to over-ground walking [14].

However, studies performed in adults cannot directly be transferred to children, as measures of kinematic variability and muscle recruitment patterns indicate that the normal gait pattern continues to develop into childhood [15-17].

Therefore, the purpose of this study was to evaluate the surface electromyography (sEMG) patterns during walking in different, but common, rehabilitation conditions in children with neuro-orthopedic gait disorders. We hypothesized that more physiological kinematics (percentage stance and swing duration) as well as muscle activation patterns could be induced by RAGT compared to (unassisted) treadmill walking. Furthermore, we hypothesized that muscle activation levels would be higher during unsupported treadmill walking compared to walking in a driven gait orthosis (DGO) while the lowest amplitudes were expected in walking in a DGO without therapist’s encouragement. In addition, we measured healthy children in an effort to differentiate whether changes in sEMG and kinematics of children with neuro-orthopedic disorders might be induced by the rehabilitation robot itself or caused by the motor impairment of the patients.

## Methods

### Participants

Ethical approval for this study was obtained from the Cantonal Ethics Committee of Zurich, Switzerland. Inclusion criteria were: (1) age between 4–18 years, (2) femur length between 0.21- 0.47 m, (3) ability to walk independently with the aid of parallel bars, (4) ability to signal fear, pain or discomfort, (5) compliance and ability to follow simple instructions, (6) no lower leg braces or orthoses at the recorded leg, which made it impossible to fixate surface electrodes, and (7) children should meet the general requirements for the training with the driven gait orthosis Lokomat® (Hocoma AG, Volketswil, Switzerland).

Our convenience sample consisted of 17 children, 9 with motor impairments (in- and outpatients of the clinic) and 8 healthy children (from local schools), who participated in the study. Parental written informed consent and child assent were achieved. The children with motor impairments had a variety of neuro-orthopedic problems that affected gait. The characteristics of the participants are displayed in Table 1.

**Table 1:** Characteristics of the children with motor impairments and the healthy controls

***Group***	***ID***	***Age***	***Gender ***	***Height (m)***	***Weight***	***Legs***	***Main diagnosis***	***Walking pattern***	***Daily life mobility aids***
		***(years)***	***(F/M)***		***(kg)***		***(GMFCS Level)***		
Patient	1	12	F	1.48	41	T	CP, spastic diplegia (II)	Diplegic gait	None
Patient	2	8	M	1.27	25	K	CP, spastic diplegia (II)	Diplegic gait	None
Patient	3	15	M	1.68	48	T	CP, spastic diplegia (II)	Diplegic gait	Bilateral ankle-foot orthosis
Patient	4	16	F	1.78	61	T	Hip dysplasia, six months post surgery	Trendelen-burg	None
Patient	5	17	F	1.61	56	T	Cerebral hemorrhage at age of 2 years	Spastic hemiplegic gait	Ankle-Foot orthosis left
Patient	6	15	F	1.68	50	T	Multiple sclerosis	Diplegic gait	Ankle-Foot orthosis left/ Underarm crutches
Patient	7	15	F	1.69	59	T	Encephalo-pathy	Diplegic gait	None
Patient	8	16	M	1.58	47	T	CP, spastic tetraplegia (III)	Crouch gait	Wheelchair
Patient	9	14	F	1.60	48	T	Transverse myelitis	Paraplegic	Ankle-Foot orthosis right/ Underarm crutches
Mean ± SD		14 ± 3		1.60 ± 0.15	48 ± 10				
Healthy	10	10	F	1.40	44.3	K	-	-	-
Healthy	11	13	M	1.54	40.0	T	-	-	-
Healthy	12	11	F	1.40	32.8	K	-	-	-
Healthy	13	9	F	1.37	32.0	K	-	-	-
Healthy	14	10	F	1.40	34.0	K	-	-	-
Healthy	15	8	M	1.43	33.9	K	-	-	-
Healthy	16	17	F	1.68	53.0	T	-	-	-
Healthy	17	16	F	1.69	64.5	T	-	-	-
Mean ± SD		12 ± 3		1.49 ± 0.13	42 ± 12				

Abbreviations: *M* male, *F* female. *K* Kids (pediatric leg module), *T* Teens (adult leg module), *CP* Cerebral Palsy; *GMFCS* Gross Motor Function Classification Scale.

GMFCS Level I = walks without restrictions but has limitations in more advanced gross motor skills.

GMFCS Level II = walks without assistive mobility device but has limitations in walking outdoor and in the community; walking stairs with hands on the handrail; running and jumping with restrictions.

GMFCS Level III = walks with assistive mobility devices but has limitations walking outdoors and in the community; walking stairs with hands on the handrail possible; depending on abilities of upper extremities independent moving with wheelchair; wheelchair for longer walking distances.

GMFCS Level IV = self mobility with limitations but is transported or uses power mobility outdoors and in the community.

Please note, Ankle-Foot orthoses were not worn during recording (see in- and exclusion criteria).

### Procedure

The study was performed at the Rehabilitation Center of the University Children’s Hospital Zurich, Switzerland. All testing started with walking in the Lokomat® followed by walking on a treadmill. This was done to ensure that electrodes could remain on the same location both in Lokomat® and treadmill walking conditions. The test protocol was part of a more extended protocol containing five different randomly presented DGO walking conditions, each lasting 2 minutes, where training with virtual realities played an important role. A more detailed description is provided elsewhere [18].

For this study, we focused on the sEMG pattern and the relative duration of the stance and swing phase of three walking conditions: (I) *DGO walking*, (II) *DGO walking motivated by therapist* and (III) *walking unassisted on a conventional treadmill*.

The participants walked at minimum 5 minutes to become familiar with the DGO before the measurements of about 12 minutes walking in the DGO started without breaks between the different conditions. Then, the DGO was removed and the participants had a 5 minutes break, before they had the possibility to become familiar with treadmill walking (at least 2 minutes). Consecutively, the recordings during 2 minutes unsupported treadmill walking were made. The therapist’s protocol to encourage and motivate the children (i.e. condition DGO with motivation) was strictly standardized and included general test instructions, specific wording, as well as the duration of constant cheering (e.g. “walk as active as you can”, “give maximal effort”, or good like this, keep it up”).

### Apparatus and tasks

#### Driven gait orthosis Lokomat®

The Lokomat® contains two actuated leg orthoses, fitted with straps on the patient’s legs. The patient’s legs are moved in a kinematically consistent, physiological walking pattern, guided by two drives installed in the hip and knee joints on each side of the exoskeleton. A weight bearing system provides bodyweight support for the patient. In this study, the amount of unloading was set to 30% of the children’s bodyweight. The treadmill velocity was synchronized with the movements of the DGO and set at the child’s comfortable walking speed. The comfortable speed averaged 1.5 km/h (0.41 m/s) for the pediatric leg module (kids’ legs: for a femur length of 0.21-0.35 m) and 1.7 km/h (0.47 m/s) for the adult leg module (teens’ legs: for a femur length of 0.35-47 m). Each participant wore passive foot lifters that provided sufficient ankle dorsiflexion for adequate toe-clearance especially during the swing phase.

#### Data recording

**Figure 1:**
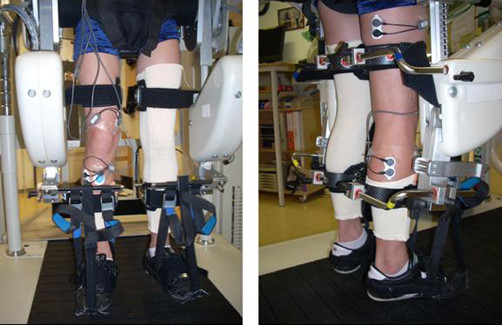
**Intervention setting.** Surface electromyography electrodes placed in the driven gait orthosis (Lokomat®) on the tibialis anterior, gastrocnemius lateralis, vastus medialis and biceps femoris muscles on one leg.

Surface electromyographic recordings were made using self-adhesive Ag/AgCl dual snap gel electrodes (Noraxon Inc, Scottsdale/USA) with a diameter of 10 mm and an inter-electrode distance of 20 mm. The electrodes were placed according to SENIAM recommendations [19,20], when permitted by the orthosis (Figure 1), over the muscle belly of four muscles on the dominant (in patients the less affected) leg: tibialis anterior (TA), gastrocnemius lateralis (GM), vastus medialis (VM) and biceps femoris (BF). The reference electrode (Blue Sensor, by Ambu) was attached overlying the tuberositas tibiae.

Data acquisition was performed with a 4-channel Myosystem 1400A (Noraxon Inc, Scottsdale/USA). Incoming signals of one leg were sampled at 2000 Hz with a pre-amplifier gain of 500 and monitored on-line, to ensure good data quality. Each recording was individually inspected after recording, as recommended by Chang et al. [21]. To gain information about the muscle activity in relation to the gait cycle, digital video recordings (50 Hz) were synchronized with the sEMG recordings. Each condition that was recorded lasted about 2 minutes.

### Data processing and statistics

Data analysis and processing was performed off-line by MyoResearch XP 1.07 Master Edition software (Noraxon Inc., Scottsdale/USA). In line with our previous publication [18], and in accordance to the SENIAM guidelines, raw sEMG signals were high-pass filtered with a bi-directional zero-lag Butterworth at cut-off frequency of 10 Hz, rectified and smoothed by Root Mean Square algorithm with a time window of 100 ms to build the linear envelope [22]. For each child, for each muscle and each condition, we calculated the average sEMG pattern of 20 strides (heel strike to toe off and toe off to heel strike) of one leg, in the middle of the two minutes recording time. Stance and swing phase were determined by hand for each step using the synchronized video recordings.

We calculated the duration of stance and swing phase as a percentage of 100% gait cycle, and the average sEMG amplitude (for each muscle and gait phase).

In order to compare similarity in averaged sEMG trajectories between conditions and groups, we calculated the average sEMG trajectory for each group (for each muscle and condition). As we also evaluated similarity in sEMG patterns for the stance and swing phase separately, we normalized the duration of the stance phase as well as the swing phase to 100% using MATLAB 7.1 software (the MathWorks, Inc. Massachusetts, USA). This was done to overcome differences in (relative) duration between stance and swing that might influence the sEMG activation patterns.

Reference sEMG patterns were obtained from a study of Chang et al. [21] with 87 healthy, normally developed children, ranging from age 3 to 18 years who walked at self-selected speed. As we were unable to derive the original data, the figures in the paper were digitized with Plot Digitizer software (http://plotdigitizer.sourceforge.net) and in addition normalized to 100 data-points using MATLAB 7.1. Here, in addition, we calculated also time-normalized reference trajectories for stance and swing phase separately to enable making correlations with our sEMG patterns.

Statistical calculations were performed with SPSS 19.0 (SPSS Inc., Illnois, USA). First, the data was checked for normal distribution with a Shapiro-Wilk test and histograms. Hence, further analysis was done with non-parametric statistics. Differences between the three conditions (DGO walking, DGO walking with motivation and walking on the treadmill) were analyzed for the duration of stance and swing phase, as well as average sEMG amplitudes (for each group and muscle separately). Within groups, a Friedman test with consecutive Wilcoxon signed rank tests and Bonferroni correction was applied. For each condition, gait phase and muscle, differences between the groups (healthy versus patients) were tested with the Mann–Whitney U test.

To compare similarity between muscle activation patterns between conditions, between groups, and with the reference trajectories, the similarity between averaged sEMG trajectories was quantified using Spearman’s correlation coefficients (ρ). The correlations were interpreted as follows (adopted from [23]): r < 0.20, poor relationship; 0.21-0.40, fair; 0.41-0.60, moderate; 0.61-0.80, good and 0.81-1.00 values very good to excellent. Alpha was set at 0.05.

## Results

### Participant’s characteristics

Children in the two groups (*with neuro*-*orthopedic gait disorders and healthy*) disclosed differences in age, height and weight, but without statistical significance (see Table 1).

### Gait cycle events: stance and swing phase distribution

**Figure 2:**
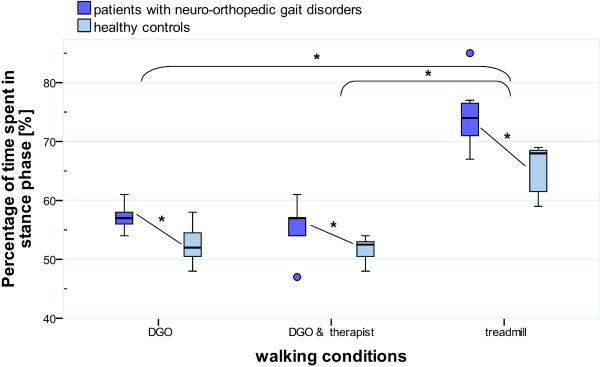
**Gait cycle event differences between groups and conditions.** Differences of time spent in stance or swing phase, sorted by groups. DGO = Driven Gait Orthosis. DGO & therapist = DGO walking with motivational therapist’s instructions. * indicate significant differences; ° are outliers.

The average percentage of total stance time in the patients was 57% ± 2% (mean ± SD) in DGO walking, 56% ± 4% in DGO with motivation and 74% ± 5% in treadmill walking. The percentage stance phase duration of the healthy participants amounted to 54% ± 3% in DGO walking, 53% ± 2% in DGO with motivation and 67% ± 4% in treadmill walking. These percentages differed not between walking in the DGO with versus without motivation (p = 0.12 for patients and p = 0.38 for healthy subjects), but the relative stance duration was significantly longer during treadmill walking compared to the DGO conditions (p = 0.012 for all comparisons; see also Figure 2). For all three conditions, however, healthy participants had a shorter relative stance phase compared to the patients (p = 0.008 for each DGO condition and p = 0.003 for treadmill walking).

### Surface electromyography amplitudes and patterns

**Figure 3:**
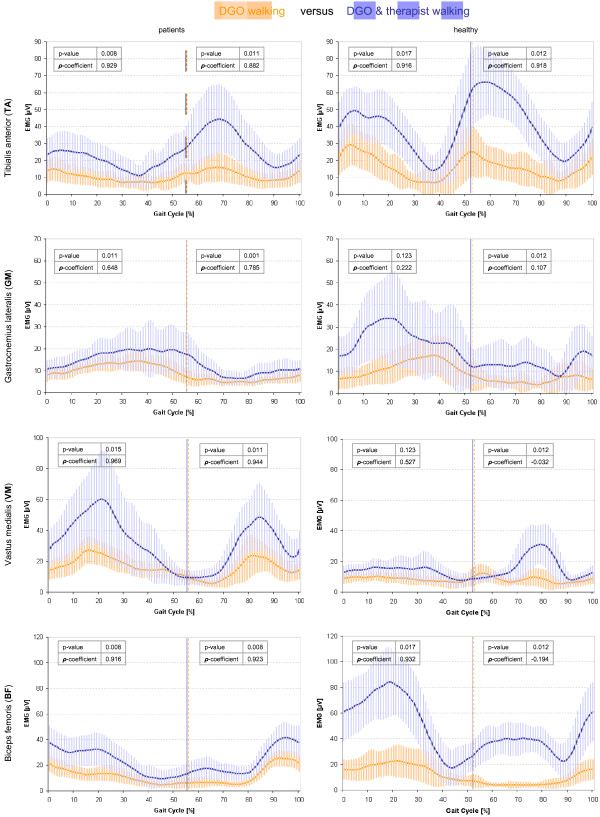
**Linear envelopes of the sEMG: comparison between DGO with and without therapeutic encouragement.** sEMG mean amplitudes of the four muscles between the different walking conditions within groups. sEMG variability is indicated with ±1SD shades in the background of the average curve. P-values as well as Spearman correlation coefficients (ρ) are displayed in the graphs. Orange = DGO walking; blue = DGO & therapist. Differences in amplitudes between the curves are presented with the p-value; correlations with the ρ-coefficients. sEMG = surface electromyography; TA = tibialis anterior; GM = gastrocnemius lateralis; VM = vastus medialis; BF = biceps femoris; DGO = Driven Gait Orthosis; DGO & therapist = DGO walking with motivational therapist’s instructions.

**Figure 4:**
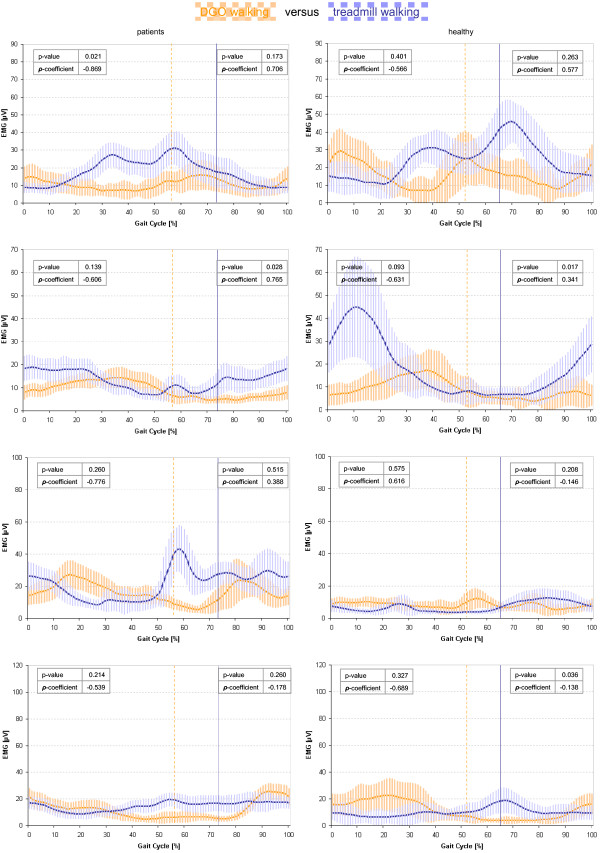
**Linear envelopes of the sEMG: comparison between walking in DGO and treadmill walking.** sEMG mean amplitudes of the four muscles between the different walking conditions within groups. sEMG variability is indicated with ±1SD shades in the background of the average curve. P-values as well as Spearman correlation coefficients (ρ) are displayed in the graphs. Orange = DGO walking; blue = treadmill walking. Differences in amplitudes between the curves are presented with the p-value; correlations with the ρ-coefficients. sEMG = surface electromyography; TA = tibialis anterior; GM = gastrocnemius lateralis; VM = vastus medialis; BF = biceps femoris; DGO = Driven Gait Orthosis.

**Figure 5:**
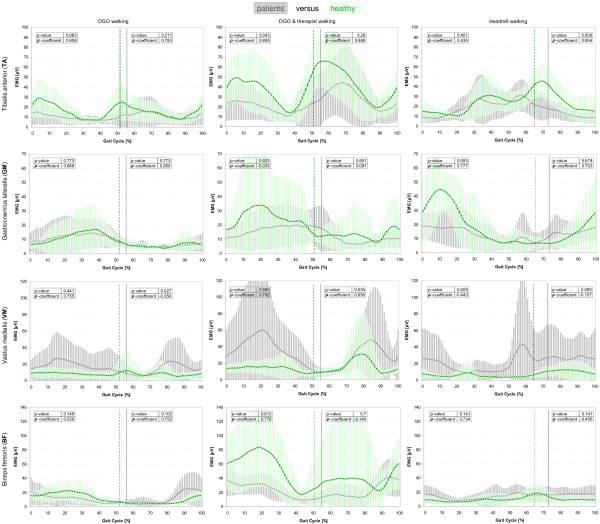
**Linear envelopes of the sEMG: comparison between patients and healthy participants.** sEMG mean amplitudes of the four muscles between the two groups within the different walking conditions. sEMG variability is indicated with ±1SD shades in the background of the average curve. P-values as well as Spearman correlation coefficients (ρ) are displayed in the graphs. Green = healthy children; grey = patients with neuro-orthopedic walking disorders. Differences in amplitudes between the curves are presented with the p-value; correlations with the ρ-coefficients. sEMG = surface electromyography; TA = tibialis anterior; GM = gastrocnemius lateralis; VM = vastus medialis; BF = biceps femoris; DGO = Driven Gait Orthosis; DGO & therapist = DGO walking with motivational therapist’s instruction.

Figures 3 and 4 illustrates the in general significantly higher sEMG amplitudes for DGO walking with therapist’s encouragement than without, even for patients as for healthy children. Figure 5 presents remarkable low between-group differences regarding the occurrence of the maximum peak (visually determined) and mean average amplitude. Within walking conditions, significant differences were observable in the TA muscle (swing phase) and the VM muscle (stance phase) during treadmill walking as well as in the BF muscle (stance phase) during DGO with motivation.

When comparing the sEMG patterns of all conditions to over-ground walking reference data from Chang et al. [21], DGO as well as DGO with motivation conditions correlated in general well to very well. On the contrary, sEMG pattern recorded during treadmill walking correlated often negatively with the reference sEMG trajectories (Table 2).

**Table 2:** Spearman rank correlation coefficient of sEMG patterns to reference patterns

***Gait cycle phase***	***Muscle***	***Healthy***			***Patient***		
		***DGO***	***DGO & therapist***	***Treadmill***	***DGO***	***DGO & therapist***	***Treadmill***
Stance	TA	0.91	0.85	- 0.44	0.90	0.84	- 0.97
	GM	0.95	0.38	- 0.48	0.95	0.82	- 0.78
	VM	0.95	0.58	0.53	0.79	0.77	- 0.48
	BF	0.52	0.69	- 0.70	0.96	0.93	- 0.56
Swing	TA	0.84	0.60	0.31	0.76	0.41	0.38
	GM	0.86	0.19	0.26	0.90	0.91	0.77
	VM	0.02	- 0.84	- 0.96	- 0.51	- 0.56	0.06
	BF	0.48	- 0.19	- 0.60	0.71	0.62	0.49

We did not perform extensive statistical analyses on these data, because the reference patterns were derived by digitizing data from Chang et al. [21]. We provided these correlation coefficients to support the visual impression one could have when comparing these sEMG trajectories.

Abbreviations: *sEMG* surface electromyography, *TA* tibialis anterior, *GM* gastrocnemius lateralis, *VM* vastus medialis, *BF* biceps femoris. *DGO* Driven Gait Orthosis.

## Discussion

The aim of this study was to compare gait phase characteristics and muscle activation patterns during different rehabilitative walking conditions (DGO walking and treadmill walking) in groups of healthy children and children with neuro-orthopedic gait disorders.

Our main findings were: (i) differences in kinematics (duration of stance and swing phase) between young patients and healthy participants were found; (ii) Muscle activation patterns of young patients with neuro-orthopedic movement disorders who walked in DGO conditions resembled reference curves of healthy children walking over-ground in general well; (iii) In addition, muscle activation patterns were quite similar between the healthy participants and the young patients during walking in the DGO; (iv) Encouragement of the therapist during DGO results in higher sEMG amplitudes compared to DGO without motivation in patients.

### Stance phase duration can be influenced during robot-assisted gait training

For persons unfamiliar with RAGT, it might come as a surprise that step duration can actually be influenced by patients and healthy participants when walking in a DGO. Despite that the device used in this study (Lokomat®), is position controlled (i.e. there is a fixed relationship between position of the legs and time during the gait cycle), differences in relative stance and swing duration were observed between healthy children and those with neuro-orthopedic diagnoses. Compared to the healthy participants, the patients had a prolonged stance phase duration. Although the relative duration of the stance phase is larger at slower speeds [24], it is unlikely that speed was the underlying factor here, as the walking speed in the DGO condition was similar for both groups (healthy participants: 1.70 ± 0.05 km/h; patients: 1.70 ± 0.20 km/h). During treadmill walking, differences in walking speed might have contributed to the difference in stance duration, as the patients walked slower (1.3 ± 0.4 km/h) compared to the healthy participants (1.7 ± 0.05 km/h).

Nevertheless, during DGO, the relative duration of the stance phase of our patients resembled already reported percentages for healthy children walking over-ground well. Granata et al. [16] showed in 11 healthy children (mean age 6.5 years) who walked over-ground that the stance duration amounted to 58.7% ± 2.6% (mean ± SD) of the gait cycle, despite a higher walking speed (4.28 ± 1.12 km/h). In addition, Chang et al. [21] showed in 26 healthy children (mean age 14.7 years) that the stance duration over-ground amounted to 60% ± 2%. During unassisted treadmill walking, patients and healthy participants spent considerably more time in the stance phase compared to these values.

### Comparing muscle activation patterns during the DGO conditions and treadmill walking with reference patterns

For clinical practice it appears important to promote exploration of movement strategies, therefore to train on restorative rather than compensatory mechanisms [25]. Furthermore, to induce functionally relevant plastic changes in the brain, training should be task-specific, because brain plasticity in human locomotor networks seems to be task-dependent as well [26]. Indeed, both treadmill walking and RAGT can be considered forms of task-dependent training.

Interestingly, in our study several results indicated that RAGT could induce a physiological walking pattern (aiming at restoration) in children with neuro-orthopedic movement disorders. When comparing our muscle activation patterns to published data from Chang et al. [21], we noticed that the DGO walking conditions correlated better to walking over-ground than treadmill walking, both in healthy children and patients. Particularly, the DGO condition without encouragement led to the most physiological muscle activation patterns. In patients, however, additional motivation of the therapist did not deteriorate the muscle activation pattern, and as muscle activation amplitudes were higher with encouragement, this condition might actually be favored when training young patients with neuro-orthopedic movement disorders. While the sEMG amplitudes during additional encouragement were higher in healthy children, the sEMG patterns correlated less well with the normal DGO walking condition (mainly in thigh muscles during swing). We assume that this might have been caused by the ability of the healthy children to generate excessive muscle strength, but at inappropriate time-points during the gait cycle against the robot. This might not have occurred in the patients, as they still required proper guidance of the movement by the DGO. Consequently, it is important to underline that the motivational instructions should not only be hortative but also specific to gait events. The smallest correlation with muscle activation patterns from healthy children walking over-ground was found for the VM muscle activity during swing. VM activity was prolonged in patients. We consider this less important, as the main function of VM is to extend and stabilize the knee during stance.

When we compared patients walking on the treadmill with the healthy over-ground reference muscle activation patterns, these patterns were substantially different, especially during the stance phase. We assume that when therapists would have manually assisted the walking pattern of patients during treadmill walking, kinematics and EMG patterns might have been more physiological. Domingo et al. [27] could observe this for adult patients with incomplete spinal cord injury, but only for the VM muscles and especially at higher speeds, whereas they mentioned there that it would be difficult for the patients anyway to walk at fast speeds without assistance. Another reason might be the increased metabolic costs for patients when walking without passive guidance [28]. Nevertheless, we also observed poor correlations for the healthy participants during treadmill walking and the healthy reference values. We are not sure what might have caused these substantial differences, as visually walking on the treadmill appeared normal. However, even healthy children are known to walk with high variability in the pattern of muscular activation [29]; within session sEMG variability in children aged 6–8 years was twice as high as reported in adults [16]. Chang et al. [21] found in children that about 13% of the sEMG curves were not functionally interpretable as physiological gait patterns. Both muscle activation patterns and stride to stride variability showed substantial variability [21] and stride to stride variability is higher in patients with neurological impairments [17]. Furthermore, walking speed was relatively slow. It is unlikely that slow speed itself might have influenced muscle activation patterns, as these remain relative stable, while the amplitudes change substantially [30]. Only for very slow walking speeds (0.06 m/s; 0.2 km/h), additional bursts can be observed [30]. However, the slow walking speed might have increased balance requirements as all children had to keep (slight) hand contact with the parallel bars next to the treadmill. Finally, all children were still wearing the harness during treadmill walking (without bodyweight support). Both factors might have influenced the walking pattern and therewith muscular activation patterns.

### Comparing muscle activation patterns during different conditions

In TA, the typical onset of activation starts before toe-off continuing with full swing phase up to heel strike and loading (about 55-15% gait cycle). We observed TA activity up to approximately 40% of the stance phase. This has been reported previously (e.g. [31]) and was explained by the activity of TA as a foot inverter muscle to control balance during single support and contra lateral limb swing [29]. Abnormal silence of the TA muscle in terminal swing was reported in patients with lengthened Achilles tendon after clubfoot surgery as well as prolonged GM muscles [32]. This effect was also visible in our study, while most physiological TA activity in late swing could be determined in both groups during DGO walking with motivation.

Nevertheless, TA activity in DGO and treadmill walking appeared more silent in the loading response and the terminal swing compared to normal. We assume that the presence of foot-lifers during DGO enabled good foot clearance during the swing phase and might have facilitated eccentric muscle control during heel strike. Except for the stance phase in patients, we could not observe a significant lower TA activity during DGO compared to treadmill walking. Lower TA activity levels in the DGO in adults have been reported previously [33] and were explained by the use of foot-lifters. We could not observe this as that much, potentially because we tried to adjust the tension of the foot-lifers to the needs of the child; enough for good foot clearance during swing, but not too strong to make the ankle joint stiff.

Normal activation of GM muscle starts at mid-swing, develops to a maximum at terminal stance and pre-swing (approximately 15-50% of the gait cycle) and is silent during swing. Our results show an early onset of GM activity during the end of swing, as well as a prolonged activity in stance. This is also known as the plantar flexion-knee extension couple to control the second rocker and an upright position. We found this especially in healthy children, mainly during treadmill walking and DGO with motivation condition. Especially in patients, GM amplitudes were small. This could be a consequence of the 30% body-weight-support during DGO walking, which might have reduced the anti-gravitational activity of already weakened GM muscles.

Best GM muscle activity pattern in our study could be found in both groups during DGO walking and in patients with neuro-orthopedic disorders also during DGO with motivation.

Normally, VM is active from mid-swing to mid-stance (75-30% gait cycle). However, during treadmill condition in our study, especially the children with neuro-orthopedic impairments showed activation in terminal stance, which might indicate a co-contraction for stabilization the knee joint before entering in pre-swing. Similar results were observed in the study of [29]. It is noticeable that the VM activity during treadmill walking was higher in children with neuro-orthopedic disorders than in the healthy children. This could be a consequence of the suboptimal gait pattern requiring higher muscle activity. This finding has also previously been reported by Lauer et al. [34], who compared rectus femoris and medial hamstrings activity between younger children and older ones, as well as between typical developed children and children with cerebral palsy. In typically developing children, older children had elevated muscle activity compared to the younger ones, while children with cerebral palsy showed much higher activity levels in the younger ones, especially in rectus femoris. Another finding was that the VM activity was very variable in the patients. Nevertheless, while the importance of quadriceps muscles is known in gait rehabilitation, it is nice to see that VM activity could be visually observed as most physiological in patients with neuro-orthopedic disorders during DGO as well as DGO with motivation conditions.

The most frequent activation modality of BF starts during mid-swing and continues up to mid-stance (85-10% gait cycle). In our results, BF was exceptionally silent in the DGO and treadmill condition, but highly activated during DGO with additional therapist motivation, mainly in late loading and mid-stance as well as in terminal swing. This could be explained by the excessive backward push of the participants’ leg after heel strike and the resistance of the DGO to this movement, which was also observable in the study of Hidler and Wall [33].

### Encouragement increases muscle activation without affecting the pattern in patients

We expected that therapeutic motivation could increase muscle activation without changing the muscle activation patterns in their shape. This could be confirmed in all four muscles during the whole gait cycle in children with neuro-orthopedic gait disorders. Similarly, healthy controls could increase muscle activation during RAGT with additional encouragement, except for GM and VM during stance. In contrast to the patients, however, the muscle activation patterns changed considerably for GM, VM and BF (the latter only during the swing phase).

### Methodological considerations

This study leaves some space for improvement in either design or data acquired. First, the number of children is relatively small and the group is relative inhomogeneous. Nevertheless, these children represent the patient population that a pediatric rehabilitation center has.

Second, although at least 2 minutes were given to familiarize the participants with the treadmill or 5 minutes for the robotic device respectively, this may have been not enough to ensure habituation and could have influenced the gait pattern.

Third, due to practical limitations in the test protocol, treadmill walking was always recorded at the end of the procedure, which might have caused some fatigue. On the one hand, this might explain why we found hardly any differences between muscle activity amplitudes between the DGO and the treadmill condition, despite that during DGO, 30% bodyweight support was provided. On the other hand, a break was provided before treadmill walking started. In addition, patients spent less time walking in the DGO compared to a regular clinical training session. Furthermore, even for the healthy participants, we found hardly any differences between the DGO and the treadmill condition. Therefore, it is unlikely that these experienced fatigue, as they walked at considerable slower speeds compared to normal. Finally, we did not investigate muscle activation patterns and kinematics during treadmill walking with therapeutic encouragement. This could also be considered a limitation of this study.

Fourth, especially during treadmill walking, it was sometimes difficult to trigger “heel strike” and “toe off”, as this was performed manually through video synchronization. Especially in these patients, the normal heel-toe gait pattern is often variable or absent, which forced us to use video-synchronization rather than foot-switches.

Fifth, while sEMG data were gathered with 2000 Hz, video recordings were made with 50 Hz only. The reduced sampling rate of the video recordings might have influenced the accuracy of determining stance and swing phase, however, due to the low walking speed of the children this is not a critical issue. Moreover, results will not be affected differently between patients and healthy participants, because both walked at equal speeds during DGO conditions, but it might have influenced the results obtained during treadmill walking.

## Conclusions

Walking in the DGO resulted in physiological muscle activation patterns for most of the muscles that we recorded (TA, GM, VM during stance and BF) in our patients with neuro-orthopedic disorders. These patterns were more physiological compared to unassisted treadmill walking, which indicates that a DGO system is able to take influence in the gait pattern of children with neuro-orthopedic gait disorders in a positive and physiological manner. For children with neuro-orthopedic disorders as assessed in this study, we recommend to combine DGO walking with therapeutic encouragement, as it results in physiological muscle activation patterns with considerable muscle activation. The resemblance with reference EMG patterns indicates that DGO training might exploit restorative mechanisms, while treadmill training might have more the aim of working on functional and compensatory processes. This might be of interest when defining patients-individual aims and goals of gait rehabilitation. Nevertheless, it is clear that this paper provides no answers on clinically important questions like the optimal dosage and intensity of training, or how well skills acquired during DGO assisted walking can be transferred to over-ground conditions in children with neuro-orthopedic disorders. Further research is necessary to demonstrate whether the advantageous prerequisites of DGO training as found in this study might actually result in improved clinical outcome.

## Abbreviations

BWSTT: Body weight supported treadmill training; RAGT: Robotic-assisted gait training; sEMG: Surface electromyography; DGO: Driven gait orthosis; TA: Tibialis anterior; GM: Gastrocnemius lateralis; VM: Vastus medialis; BF: Biceps femoris.

## Competing interests

The authors declare that they have no competing interests and there are not any financial competing interests do declare in relation to this manuscript.

## Authors’ contributions

TAS was responsible for study design, subject recruitment, data acquisition, complete data analysis/interpretation and writing the first draft. RM and HvH made contributions to the methodology and data interpretation and carefully reviewed the manuscript. All authors approved the final manuscript.
